# 1-(2,6-Dichloro­benzo­yl)-3-(2,3,5,6-tetra­chloro­phen­yl)thio­urea trichloro­methane hemisolvate

**DOI:** 10.1107/S1600536809000051

**Published:** 2009-01-08

**Authors:** M. Khawar Rauf, Michael Bolte, Saeed Anwar

**Affiliations:** a Department of Chemistry, Quaid-i-Azam University, Islamabad 45320, Pakistan; b Institut für Anorganische Chemie, J. W. Goethe-Universität Frankfurt, Max-von-Laue-Strasse 7, 60438 Frankfurt/Main, Germany; c Department of Chemistry, Islamia College University, Peshawar, Pakistan

## Abstract

The title compound, C_14_H_6_Cl_6_N_2_OS·0.5CHCl_3_, crystallizes with four 1-(2,6-dichloro­benzo­yl)-3-(2,3,5,6-tetra­chloro­phen­yl)thio­urea mol­ecules and two trichloro­methane mol­ecules in the asymmetric unit. The thiourea molecules exist in the solid state in their thione forms with typical thio­urea C—S and C—O bonds lengths, as well as shortened C—N bonds. The —NH—C(=S)—NH—C(=O)— plane is almost perpen­dicular to the benzene ring in each thiourea molecule. Intra­molecular N—H⋯O hydrogen bonds stabilize the mol­ecular conformation and inter­molecular N—H⋯S hydrogen bonds stabilize the packing arrangement.

## Related literature

For related compounds, see: Khawar Rauf *et al.* (2006*a* ▶,*b* ▶, 2007 ▶). For standard bond-length data, see: Allen (2002 ▶).
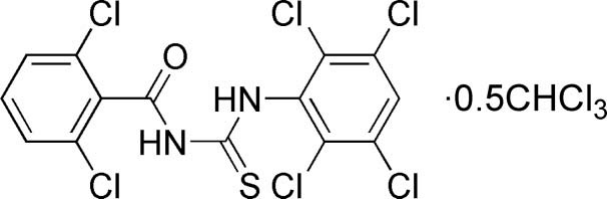


## Experimental

### 

#### Crystal data

C_14_H_6_Cl_6_N_2_OS·0.5CHCl_3_
*M* *_r_* = 522.65
Monoclinic, 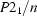

*a* = 26.7213 (6) Å
*b* = 8.6580 (2) Å
*c* = 36.1046 (9) Å
β = 110.683 (2)°
*V* = 7814.6 (3) Å^3^
*Z* = 16
Mo *K*α radiation
μ = 1.20 mm^−1^
*T* = 173 (2) K
0.43 × 0.41 × 0.38 mm

#### Data collection

Stoe IPDS II two-circle diffractometer
Absorption correction: multi-scan (*MULABS*; Spek, 2003 ▶; Blessing, 1995 ▶) *T*_min_ = 0.627, *T*_max_ = 0.659
156440 measured reflections
22452 independent reflections
17143 reflections with *I* > 2σ(*I*)
*R*_int_ = 0.052

#### Refinement

*R*[*F* ^2^ > 2σ(*F* ^2^)] = 0.051
*wR*(*F* ^2^) = 0.147
*S* = 1.08
22452 reflections
969 parameters
H atoms treated by a mixture of independent and constrained refinement
Δρ_max_ = 1.64 e Å^−3^
Δρ_min_ = −1.40 e Å^−3^

### 

Data collection: *X-AREA* (Stoe & Cie, 2001 ▶); cell refinement: *X-AREA*; data reduction: *X-AREA*; program(s) used to solve structure: *SHELXS97* (Sheldrick, 2008 ▶); program(s) used to refine structure: *SHELXL97* (Sheldrick, 2008 ▶); molecular graphics: *XP* in *SHELXTL-Plus* (Sheldrick, 2008 ▶); software used to prepare material for publication: *SHELXL97*.

## Supplementary Material

Crystal structure: contains datablocks I, global. DOI: 10.1107/S1600536809000051/ez2154sup1.cif

Structure factors: contains datablocks I. DOI: 10.1107/S1600536809000051/ez2154Isup2.hkl

Additional supplementary materials:  crystallographic information; 3D view; checkCIF report

## Figures and Tables

**Table 1:** Hydrogen-bond geometry (Å, °)

*D*—H⋯*A*	*D*—H	H⋯*A*	*D*⋯*A*	*D*—H⋯*A*
N1—H1⋯S1*B*	0.80 (3)	2.60 (3)	3.362 (2)	160 (3)
N2—H2⋯O1	0.78 (3)	1.96 (3)	2.621 (2)	142 (3)
N2—H2⋯Cl4*C*^i^	0.78 (3)	2.94 (3)	3.539 (2)	135 (3)
N1*A*—H1*A*⋯S1*C*	0.84 (3)	2.55 (3)	3.381 (2)	170 (3)
N2*A*—H2*A*⋯O1*A*	0.75 (3)	2.00 (3)	2.625 (3)	141 (3)
N2*A*—H2*A*⋯Cl4*A*^ii^	0.75 (3)	2.90 (3)	3.478 (2)	136 (3)
N1*B*—H1*B*⋯S1	0.83 (3)	2.63 (3)	3.425 (2)	162 (3)
N2*B*—H2*B*⋯O1*B*	0.78 (3)	2.01 (3)	2.639 (2)	138 (3)
N1*C*—H1*C*⋯S1*A*	0.86 (3)	2.57 (3)	3.416 (2)	168 (3)
N2*C*—H2*C*⋯O1*C*	0.77 (3)	1.97 (3)	2.629 (3)	144 (3)

Symmetry codes: (i) 
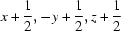
; (ii) 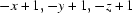.

**Fig. 1.**
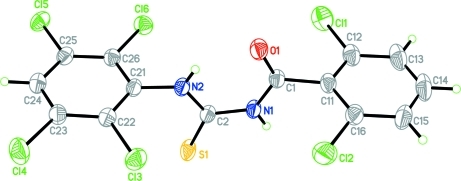
Molecular structure of (I) showing atom numbering scheme. Displacement ellipsoids are drawn at the 50% probability level.

**Fig. 2.**
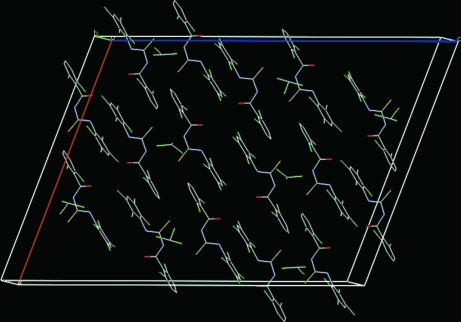
Packing diagram of (I) with a view onto the ac plane. H atoms omitted for clarity.

## supplementary crystallographic information

### Comment

The background to this study has been set out in our previous work on the structural chemistry of *N*,*N'*-disubstituted thioureas (Khawar Rauf *et al.*, 2006*a*, 2007). Herein, as a continuation of these studies, the structure of the title compound (I) is described. A depiction of the molecule is given in Fig. 1. Bond lengths and angles can be regarded as typical for *N*,*N'*-disubstituted thiourea compounds as found in the Cambridge Structural Database v5.28 (Allen, 2002; Khawar Rauf *et al.*, 2006*b*). The molecule exists in the thione form with typical thiourea C—S and C—O bonds, as well as shortened C—N bond lengths. The thiocarbonyl and carbonyl groups are almost coplanar. The molecule features an intramolecular N—H···O hydrogen bond (see the table of hydrogen bond geometry; Fig 2).

### Experimental

Freshly prepared and steam distillated 2,6-dichlorobenzoyl isothiocyanate (2.32 g, 10 mmol) was stirred in acetone (50 ml) for 20 minutes. Neat 2,3,5,6-tetrachloroaniline (2.30 g, 10 mmol) was then added and the resulting mixture was stirred for 1 h. The reaction mixture was then poured into acidified (pH 4) water and stirred well. The solid product was separated and washed with deionized water and purified by recrystallization from methanol–1,1-dichloromethane (1:10 *v/v*) to give fine crystals of (I), with an overall yield of 85%.

### Refinement

H atoms bonded to C were included in calculated positions and refined as riding on their parent C atom with C—H = 0.95 Å *U*_iso_(H) = 1.2*U*_eq_(C). The H atoms bonded to N were freely refined. The highest peak in the final difference map (1.64 e Å^-3^) is located at 1.04 Å from Cl13 and the deepest hole (-1.40 e Å^-3^) is located at 0.75 Å from Cl22.

### Crystal data

**Table:**

C_14_H_6_Cl_6_N_2_OS·0.5CHCl_3_	*F*(000) = 4144
*M**_r_* = 522.65	*D*_x_ = 1.777 Mg m^−^^3^
Monoclinic, *P*2_1_/*n*	Mo *K*α radiation, λ = 0.71073 Å
Hall symbol: -P 2yn	Cell parameters from 111596 reflections
*a* = 26.7213 (6) Å	θ = 1.6–30.3°
*b* = 8.6580 (2) Å	µ = 1.20 mm^−^^1^
*c* = 36.1046 (9) Å	*T* = 173 K
β = 110.683 (2)°	Block, colourless
*V* = 7814.6 (3) Å^3^	0.43 × 0.41 × 0.38 mm
*Z* = 16	

### Data collection

**Table:**

Stoe IPDS II two-circle diffractometer	22452 independent reflections
Radiation source: fine-focus sealed tube	17143 reflections with *I* > 2σ(*I*)
graphite	*R*_int_ = 0.052
ω scans	θ_max_ = 29.9°, θ_min_ = 1.6°
Absorption correction: multi-scan (*MULABS*; Spek, 2003; Blessing, 1995)	*h* = −37→37
*T*_min_ = 0.627, *T*_max_ = 0.659	*k* = −12→12
156440 measured reflections	*l* = −50→50

### Refinement

**Table:**

Refinement on *F*^2^	Primary atom site location: structure-invariant direct methods
Least-squares matrix: full	Secondary atom site location: difference Fourier map
*R*[*F*^2^ > 2σ(*F*^2^)] = 0.051	Hydrogen site location: inferred from neighbouring sites
*wR*(*F*^2^) = 0.147	H atoms treated by a mixture of independent and constrained refinement
*S* = 1.08	*w* = 1/[σ^2^(*F*_o_^2^) + (0.0852*P*)^2^ + 3.006*P*] where *P* = (*F*_o_^2^ + 2*F*_c_^2^)/3
22452 reflections	(Δ/σ)_max_ = 0.001
969 parameters	Δρ_max_ = 1.64 e Å^−^^3^
0 restraints	Δρ_min_ = −1.40 e Å^−^^3^

### Special details

**Table:**

Geometry. All e.s.d.'s (except the e.s.d. in the dihedral angle between two l.s. planes) are estimated using the full covariance matrix. The cell e.s.d.'s are taken into account individually in the estimation of e.s.d.'s in distances, angles and torsion angles; correlations between e.s.d.'s in cell parameters are only used when they are defined by crystal symmetry. An approximate (isotropic) treatment of cell e.s.d.'s is used for estimating e.s.d.'s involving l.s. planes.
Refinement. Refinement of *F*^2^ against ALL reflections. The weighted *R*-factor *wR* and goodness of fit *S* are based on *F*^2^, conventional *R*-factors *R* are based on *F*, with *F* set to zero for negative *F*^2^. The threshold expression of *F*^2^ > σ(*F*^2^) is used only for calculating *R*-factors(gt) *etc*. and is not relevant to the choice of reflections for refinement. *R*-factors based on *F*^2^ are statistically about twice as large as those based on *F*, and *R*- factors based on ALL data will be even larger.

### Fractional atomic coordinates and isotropic or equivalent isotropic displacement parameters (Å^2^)

**Table:**

	*x*	*y*	*z*	*U*_iso_*/*U*_eq_	
Cl1	0.66337 (3)	−0.02992 (9)	0.44477 (2)	0.04817 (16)	
Cl2	0.70980 (3)	0.57423 (8)	0.47582 (3)	0.05017 (17)	
Cl3	0.89534 (3)	0.45616 (7)	0.58259 (2)	0.03868 (14)	
Cl4	0.99807 (3)	0.38781 (8)	0.65838 (2)	0.04692 (16)	
Cl5	0.96884 (3)	−0.21933 (7)	0.632157 (19)	0.03678 (13)	
Cl6	0.86205 (2)	−0.15449 (7)	0.559103 (19)	0.03543 (12)	
C1	0.73240 (8)	0.2335 (3)	0.49034 (6)	0.0269 (4)	
O1	0.73085 (7)	0.2042 (2)	0.52283 (5)	0.0355 (4)	
N1	0.77829 (7)	0.2323 (2)	0.48119 (6)	0.0271 (4)	
H1	0.7775 (13)	0.251 (4)	0.4593 (10)	0.042 (9)*	
C2	0.83002 (9)	0.2035 (3)	0.50686 (7)	0.0269 (4)	
S1	0.88115 (2)	0.20537 (9)	0.490438 (19)	0.03819 (15)	
N2	0.83492 (8)	0.1760 (2)	0.54445 (6)	0.0270 (4)	
H2	0.8084 (14)	0.177 (4)	0.5489 (10)	0.048 (10)*	
C11	0.68259 (9)	0.2763 (3)	0.45622 (7)	0.0306 (5)	
C12	0.64776 (10)	0.1631 (3)	0.43414 (7)	0.0367 (5)	
C13	0.60002 (11)	0.2023 (4)	0.40395 (8)	0.0483 (7)	
H13	0.5767	0.1242	0.3889	0.058*	
C14	0.58724 (11)	0.3558 (5)	0.39636 (8)	0.0512 (8)	
H14	0.5548	0.3828	0.3758	0.061*	
C15	0.62035 (11)	0.4715 (4)	0.41784 (9)	0.0468 (7)	
H15	0.6109	0.5771	0.4124	0.056*	
C16	0.66784 (10)	0.4306 (3)	0.44760 (8)	0.0372 (5)	
C21	0.88435 (8)	0.1473 (3)	0.57509 (6)	0.0264 (4)	
C22	0.91506 (9)	0.2696 (3)	0.59669 (7)	0.0291 (4)	
C23	0.96117 (9)	0.2387 (3)	0.62935 (7)	0.0313 (5)	
C24	0.97723 (9)	0.0884 (3)	0.63984 (7)	0.0320 (5)	
H24	1.0087	0.0685	0.6620	0.038*	
C25	0.94752 (9)	−0.0332 (3)	0.61811 (7)	0.0288 (4)	
C26	0.90057 (9)	−0.0050 (3)	0.58587 (7)	0.0275 (4)	
Cl1A	0.33429 (3)	1.13982 (9)	0.35021 (3)	0.0584 (2)	
Cl2A	0.29196 (3)	0.53262 (10)	0.32256 (2)	0.05094 (18)	
Cl3A	0.48739 (2)	0.40796 (7)	0.435585 (18)	0.03423 (12)	
Cl4A	0.59504 (2)	0.34253 (7)	0.507834 (18)	0.03507 (13)	
Cl5A	0.62697 (3)	0.94952 (8)	0.53263 (2)	0.04705 (16)	
Cl6A	0.52213 (3)	1.01845 (7)	0.45831 (2)	0.03947 (14)	
C1A	0.35869 (9)	0.8016 (3)	0.36676 (7)	0.0277 (4)	
O1A	0.35767 (7)	0.7870 (2)	0.40003 (5)	0.0376 (4)	
N1A	0.40400 (7)	0.7872 (2)	0.35718 (6)	0.0260 (4)	
H1A	0.3987 (11)	0.797 (3)	0.3331 (9)	0.029 (7)*	
C2A	0.45572 (8)	0.7577 (3)	0.38303 (6)	0.0260 (4)	
S1A	0.50641 (2)	0.74921 (8)	0.366168 (17)	0.03312 (13)	
N2A	0.46098 (8)	0.7394 (2)	0.42084 (6)	0.0276 (4)	
H2A	0.4358 (13)	0.744 (4)	0.4256 (10)	0.043 (9)*	
C11A	0.30878 (9)	0.8396 (3)	0.33229 (7)	0.0329 (5)	
C12A	0.29322 (10)	0.9926 (4)	0.32277 (8)	0.0420 (6)	
C13A	0.24564 (12)	1.0307 (5)	0.29284 (10)	0.0568 (9)	
H13A	0.2358	1.1355	0.2866	0.068*	
C14A	0.21276 (12)	0.9113 (5)	0.27230 (9)	0.0626 (11)	
H14A	0.1799	0.9357	0.2519	0.075*	
C15A	0.22641 (11)	0.7601 (5)	0.28069 (9)	0.0565 (9)	
H15A	0.2035	0.6802	0.2662	0.068*	
C16A	0.27470 (10)	0.7241 (4)	0.31097 (8)	0.0401 (6)	
C21A	0.51069 (8)	0.7098 (3)	0.45135 (6)	0.0256 (4)	
C22A	0.52656 (8)	0.5578 (3)	0.46208 (6)	0.0259 (4)	
C23A	0.57383 (9)	0.5290 (3)	0.49375 (7)	0.0280 (4)	
C24A	0.60455 (9)	0.6499 (3)	0.51502 (7)	0.0307 (5)	
H24A	0.6365	0.6294	0.5367	0.037*	
C25A	0.58857 (9)	0.8013 (3)	0.50456 (7)	0.0314 (5)	
C26A	0.54184 (9)	0.8319 (3)	0.47255 (7)	0.0294 (4)	
Cl1B	0.95781 (3)	0.58907 (8)	0.42725 (2)	0.04137 (14)	
Cl2B	0.91397 (3)	−0.01830 (8)	0.39847 (2)	0.04339 (15)	
Cl3B	0.75253 (2)	0.00790 (7)	0.312422 (18)	0.03588 (13)	
Cl4B	0.64711 (3)	−0.04636 (8)	0.238690 (19)	0.03914 (14)	
Cl5B	0.62441 (3)	0.56373 (8)	0.214061 (19)	0.04155 (15)	
Cl6B	0.73026 (2)	0.62207 (7)	0.287247 (18)	0.03314 (12)	
C1B	0.88859 (8)	0.3185 (3)	0.38354 (7)	0.0284 (4)	
O1B	0.88943 (7)	0.3517 (3)	0.35104 (5)	0.0415 (4)	
N1B	0.84291 (7)	0.3071 (2)	0.39269 (6)	0.0260 (4)	
H1B	0.8450 (11)	0.289 (3)	0.4157 (9)	0.032 (7)*	
C2B	0.79047 (8)	0.3128 (2)	0.36654 (6)	0.0246 (4)	
S1B	0.73988 (2)	0.29118 (8)	0.383021 (17)	0.03172 (12)	
N2B	0.78424 (7)	0.3360 (2)	0.32837 (5)	0.0255 (4)	
H2B	0.8096 (14)	0.356 (4)	0.3235 (10)	0.047 (9)*	
C11B	0.93940 (8)	0.2807 (3)	0.41760 (7)	0.0283 (4)	
C12B	0.97398 (9)	0.3963 (3)	0.43896 (7)	0.0325 (5)	
C13B	1.02204 (10)	0.3602 (4)	0.46905 (8)	0.0408 (6)	
H13B	1.0453	0.4396	0.4835	0.049*	
C14B	1.03531 (10)	0.2061 (4)	0.47748 (8)	0.0430 (6)	
H14B	1.0678	0.1808	0.4982	0.052*	
C15B	1.00233 (10)	0.0880 (4)	0.45648 (8)	0.0389 (6)	
H15B	1.0120	−0.0171	0.4624	0.047*	
C16B	0.95474 (9)	0.1279 (3)	0.42664 (7)	0.0325 (5)	
C21B	0.73496 (8)	0.3127 (3)	0.29691 (6)	0.0255 (4)	
C22B	0.71628 (9)	0.1617 (3)	0.28614 (7)	0.0275 (4)	
C23B	0.66976 (9)	0.1384 (3)	0.25347 (7)	0.0300 (4)	
C24B	0.64140 (9)	0.2618 (3)	0.23173 (7)	0.0331 (5)	
H24B	0.6094	0.2445	0.2097	0.040*	
C25B	0.65990 (9)	0.4111 (3)	0.24221 (7)	0.0307 (5)	
C26B	0.70672 (9)	0.4370 (3)	0.27466 (6)	0.0272 (4)	
Cl1C	0.57911 (3)	1.09097 (10)	0.30254 (3)	0.0564 (2)	
Cl2C	0.53972 (4)	0.48383 (10)	0.27287 (3)	0.0648 (2)	
Cl3C	0.38248 (2)	0.50322 (6)	0.186926 (18)	0.03258 (12)	
Cl4C	0.27591 (2)	0.43823 (7)	0.113810 (18)	0.03400 (12)	
Cl5C	0.24486 (3)	1.04537 (8)	0.08911 (2)	0.04316 (15)	
Cl6C	0.34925 (3)	1.11411 (7)	0.162963 (18)	0.03610 (13)	
C1C	0.51367 (8)	0.8182 (3)	0.25740 (7)	0.0283 (4)	
O1C	0.51427 (7)	0.8408 (3)	0.22432 (5)	0.0401 (4)	
N1C	0.46813 (7)	0.8199 (2)	0.26692 (6)	0.0259 (4)	
H1C	0.4729 (11)	0.807 (3)	0.2916 (9)	0.031 (7)*	
C2C	0.41553 (8)	0.8246 (2)	0.24069 (6)	0.0243 (4)	
S1C	0.36491 (2)	0.81802 (8)	0.257634 (17)	0.03250 (13)	
N2C	0.40942 (8)	0.8333 (2)	0.20232 (6)	0.0267 (4)	
H2C	0.4359 (12)	0.842 (3)	0.1987 (9)	0.031 (7)*	
C11C	0.56415 (9)	0.7834 (3)	0.29148 (7)	0.0327 (5)	
C12C	0.59717 (10)	0.9001 (4)	0.31357 (8)	0.0436 (7)	
C13C	0.64558 (12)	0.8641 (6)	0.34403 (9)	0.0632 (11)	
H13C	0.6680	0.9437	0.3592	0.076*	
C14C	0.65995 (12)	0.7110 (7)	0.35156 (10)	0.0773 (15)	
H14C	0.6926	0.6861	0.3721	0.093*	
C15C	0.62825 (13)	0.5938 (5)	0.33010 (11)	0.0668 (12)	
H15C	0.6389	0.4891	0.3356	0.080*	
C16C	0.58052 (11)	0.6305 (4)	0.30025 (9)	0.0450 (7)	
C21C	0.35997 (8)	0.8054 (3)	0.17128 (6)	0.0244 (4)	
C22C	0.34374 (8)	0.6527 (2)	0.16039 (6)	0.0251 (4)	
C23C	0.29691 (9)	0.6247 (3)	0.12831 (7)	0.0276 (4)	
C24C	0.26655 (9)	0.7460 (3)	0.10667 (7)	0.0300 (4)	
H24C	0.2348	0.7258	0.0847	0.036*	
C25C	0.28269 (9)	0.8969 (3)	0.11721 (7)	0.0296 (4)	
C26C	0.32928 (9)	0.9273 (3)	0.14963 (6)	0.0269 (4)	
C1L	0.82936 (12)	0.7588 (4)	0.43119 (9)	0.0493 (7)	
H1L	0.8633	0.8197	0.4391	0.059*	
Cl11	0.81304 (3)	0.70372 (9)	0.38139 (2)	0.04810 (17)	
Cl12	0.84087 (3)	0.59360 (9)	0.46202 (2)	0.04500 (15)	
Cl13	0.78014 (5)	0.87597 (12)	0.43717 (4)	0.0790 (3)	
C2L	0.4292 (3)	0.2482 (6)	0.30034 (16)	0.119 (2)	
H2L	0.3916	0.2133	0.2950	0.143*	
Cl21	0.46414 (3)	0.12027 (9)	0.33740 (2)	0.04996 (17)	
Cl22	0.42585 (6)	0.42989 (12)	0.31435 (4)	0.0903 (4)	
Cl23	0.43463 (5)	0.21729 (10)	0.25600 (3)	0.0710 (3)	

### Atomic displacement parameters (Å^2^)

**Table:**

	*U*^11^	*U*^22^	*U*^33^	*U*^12^	*U*^13^	*U*^23^
Cl1	0.0458 (4)	0.0489 (4)	0.0468 (4)	−0.0022 (3)	0.0126 (3)	−0.0124 (3)
Cl2	0.0427 (3)	0.0391 (3)	0.0703 (5)	0.0037 (3)	0.0219 (3)	0.0085 (3)
Cl3	0.0428 (3)	0.0285 (3)	0.0403 (3)	0.0043 (2)	0.0092 (3)	0.0039 (2)
Cl4	0.0430 (3)	0.0405 (3)	0.0435 (4)	−0.0040 (3)	−0.0018 (3)	−0.0083 (3)
Cl5	0.0414 (3)	0.0333 (3)	0.0370 (3)	0.0116 (2)	0.0154 (2)	0.0100 (2)
Cl6	0.0358 (3)	0.0322 (3)	0.0368 (3)	−0.0028 (2)	0.0111 (2)	−0.0031 (2)
C1	0.0243 (9)	0.0323 (11)	0.0230 (10)	0.0024 (8)	0.0072 (8)	0.0006 (8)
O1	0.0277 (8)	0.0544 (11)	0.0250 (8)	0.0026 (7)	0.0099 (6)	0.0046 (7)
N1	0.0220 (8)	0.0381 (10)	0.0211 (9)	0.0050 (7)	0.0073 (7)	0.0046 (7)
C2	0.0260 (10)	0.0301 (10)	0.0242 (10)	0.0050 (8)	0.0082 (8)	0.0038 (8)
S1	0.0246 (3)	0.0632 (4)	0.0285 (3)	0.0111 (3)	0.0114 (2)	0.0136 (3)
N2	0.0225 (8)	0.0366 (10)	0.0218 (9)	0.0042 (7)	0.0078 (7)	0.0051 (7)
C11	0.0228 (9)	0.0454 (13)	0.0242 (10)	0.0052 (9)	0.0091 (8)	0.0044 (9)
C12	0.0275 (11)	0.0558 (16)	0.0272 (12)	0.0039 (10)	0.0099 (9)	0.0002 (11)
C13	0.0274 (12)	0.084 (2)	0.0286 (13)	0.0037 (13)	0.0042 (10)	−0.0025 (13)
C14	0.0268 (12)	0.095 (3)	0.0287 (13)	0.0138 (14)	0.0062 (10)	0.0135 (14)
C15	0.0332 (12)	0.071 (2)	0.0406 (15)	0.0213 (13)	0.0177 (11)	0.0259 (14)
C16	0.0278 (11)	0.0503 (15)	0.0360 (13)	0.0078 (10)	0.0144 (10)	0.0129 (11)
C21	0.0240 (9)	0.0317 (11)	0.0225 (10)	0.0035 (8)	0.0070 (8)	0.0039 (8)
C22	0.0289 (10)	0.0300 (11)	0.0271 (11)	0.0020 (8)	0.0082 (8)	0.0023 (8)
C23	0.0284 (10)	0.0323 (11)	0.0297 (11)	0.0007 (8)	0.0060 (9)	0.0003 (9)
C24	0.0293 (10)	0.0367 (12)	0.0271 (11)	0.0039 (9)	0.0065 (9)	0.0018 (9)
C25	0.0292 (10)	0.0328 (11)	0.0255 (10)	0.0054 (8)	0.0111 (8)	0.0037 (8)
C26	0.0272 (10)	0.0301 (10)	0.0256 (10)	0.0017 (8)	0.0098 (8)	0.0006 (8)
Cl1A	0.0528 (4)	0.0425 (4)	0.0862 (6)	0.0098 (3)	0.0325 (4)	0.0141 (4)
Cl2A	0.0479 (4)	0.0557 (4)	0.0491 (4)	−0.0063 (3)	0.0171 (3)	−0.0151 (3)
Cl3A	0.0348 (3)	0.0318 (3)	0.0345 (3)	−0.0036 (2)	0.0103 (2)	−0.0033 (2)
Cl4A	0.0396 (3)	0.0327 (3)	0.0343 (3)	0.0103 (2)	0.0150 (2)	0.0092 (2)
Cl5A	0.0457 (3)	0.0398 (3)	0.0408 (3)	−0.0070 (3)	−0.0031 (3)	−0.0068 (3)
Cl6A	0.0448 (3)	0.0279 (3)	0.0401 (3)	0.0040 (2)	0.0080 (3)	0.0034 (2)
C1A	0.0247 (9)	0.0333 (11)	0.0234 (10)	0.0035 (8)	0.0063 (8)	0.0013 (8)
O1A	0.0277 (8)	0.0605 (12)	0.0253 (8)	0.0073 (8)	0.0103 (7)	0.0046 (8)
N1A	0.0225 (8)	0.0352 (10)	0.0192 (9)	0.0048 (7)	0.0061 (7)	0.0031 (7)
C2A	0.0251 (9)	0.0280 (10)	0.0226 (10)	0.0041 (8)	0.0056 (8)	0.0033 (8)
S1A	0.0240 (2)	0.0498 (3)	0.0259 (3)	0.0083 (2)	0.0092 (2)	0.0073 (2)
N2A	0.0222 (8)	0.0387 (10)	0.0207 (9)	0.0037 (7)	0.0063 (7)	0.0039 (7)
C11A	0.0229 (10)	0.0525 (15)	0.0242 (11)	0.0085 (9)	0.0094 (8)	0.0066 (10)
C12A	0.0316 (12)	0.0587 (17)	0.0413 (14)	0.0139 (11)	0.0198 (11)	0.0183 (13)
C13A	0.0412 (15)	0.088 (3)	0.0486 (17)	0.0324 (16)	0.0253 (14)	0.0369 (17)
C14A	0.0297 (13)	0.126 (3)	0.0297 (14)	0.0210 (17)	0.0080 (11)	0.0198 (18)
C15A	0.0277 (12)	0.110 (3)	0.0282 (14)	0.0069 (15)	0.0052 (10)	−0.0055 (16)
C16A	0.0259 (11)	0.0652 (18)	0.0286 (12)	0.0050 (11)	0.0089 (9)	−0.0015 (12)
C21A	0.0243 (9)	0.0314 (11)	0.0197 (9)	0.0028 (8)	0.0060 (8)	0.0020 (8)
C22A	0.0256 (9)	0.0309 (10)	0.0209 (10)	0.0006 (8)	0.0079 (8)	0.0011 (8)
C23A	0.0269 (10)	0.0322 (11)	0.0242 (10)	0.0043 (8)	0.0083 (8)	0.0040 (8)
C24A	0.0263 (10)	0.0368 (12)	0.0253 (11)	0.0027 (9)	0.0044 (8)	0.0030 (9)
C25A	0.0290 (10)	0.0328 (11)	0.0285 (11)	−0.0011 (9)	0.0051 (9)	0.0000 (9)
C26A	0.0294 (10)	0.0304 (11)	0.0261 (11)	0.0028 (8)	0.0069 (8)	0.0016 (8)
Cl1B	0.0377 (3)	0.0419 (3)	0.0425 (3)	−0.0017 (2)	0.0118 (3)	−0.0043 (3)
Cl2B	0.0402 (3)	0.0387 (3)	0.0488 (4)	−0.0030 (3)	0.0127 (3)	0.0018 (3)
Cl3B	0.0396 (3)	0.0296 (3)	0.0351 (3)	0.0034 (2)	0.0090 (2)	0.0017 (2)
Cl4B	0.0449 (3)	0.0378 (3)	0.0347 (3)	−0.0121 (2)	0.0139 (3)	−0.0110 (2)
Cl5B	0.0358 (3)	0.0440 (3)	0.0344 (3)	0.0054 (2)	−0.0006 (2)	0.0089 (3)
Cl6B	0.0352 (3)	0.0300 (3)	0.0312 (3)	−0.0020 (2)	0.0080 (2)	0.0011 (2)
C1B	0.0230 (9)	0.0377 (12)	0.0232 (10)	−0.0002 (8)	0.0064 (8)	0.0018 (8)
O1B	0.0260 (8)	0.0725 (14)	0.0246 (8)	−0.0039 (8)	0.0073 (7)	0.0081 (8)
N1B	0.0226 (8)	0.0345 (10)	0.0194 (9)	0.0014 (7)	0.0054 (7)	0.0021 (7)
C2B	0.0226 (9)	0.0274 (10)	0.0219 (10)	0.0011 (7)	0.0056 (7)	0.0009 (8)
S1B	0.0221 (2)	0.0493 (3)	0.0234 (3)	0.0031 (2)	0.0076 (2)	0.0035 (2)
N2B	0.0219 (8)	0.0333 (9)	0.0196 (8)	−0.0011 (7)	0.0051 (7)	0.0018 (7)
C11B	0.0204 (9)	0.0420 (12)	0.0219 (10)	0.0007 (8)	0.0065 (8)	0.0014 (9)
C12B	0.0247 (10)	0.0442 (13)	0.0278 (11)	−0.0001 (9)	0.0083 (9)	−0.0019 (9)
C13B	0.0259 (11)	0.0624 (17)	0.0300 (13)	−0.0027 (11)	0.0049 (9)	−0.0043 (12)
C14B	0.0247 (11)	0.0710 (19)	0.0282 (12)	0.0064 (11)	0.0030 (9)	0.0071 (12)
C15B	0.0316 (11)	0.0540 (16)	0.0319 (13)	0.0100 (11)	0.0121 (10)	0.0126 (11)
C16B	0.0275 (10)	0.0423 (13)	0.0287 (11)	0.0011 (9)	0.0112 (9)	0.0044 (9)
C21B	0.0235 (9)	0.0311 (10)	0.0199 (9)	−0.0009 (8)	0.0052 (8)	0.0003 (8)
C22B	0.0285 (10)	0.0303 (10)	0.0231 (10)	−0.0011 (8)	0.0085 (8)	−0.0011 (8)
C23B	0.0300 (10)	0.0347 (11)	0.0249 (11)	−0.0038 (9)	0.0092 (9)	−0.0036 (9)
C24B	0.0266 (10)	0.0438 (13)	0.0241 (11)	−0.0034 (9)	0.0032 (8)	−0.0022 (9)
C25B	0.0266 (10)	0.0377 (12)	0.0254 (11)	0.0009 (9)	0.0060 (8)	0.0022 (9)
C26B	0.0267 (10)	0.0317 (11)	0.0222 (10)	−0.0006 (8)	0.0073 (8)	0.0002 (8)
Cl1C	0.0552 (4)	0.0615 (5)	0.0609 (5)	−0.0207 (4)	0.0309 (4)	−0.0264 (4)
Cl2C	0.0592 (5)	0.0416 (4)	0.1051 (8)	0.0033 (3)	0.0433 (5)	0.0134 (4)
Cl3C	0.0338 (3)	0.0281 (3)	0.0340 (3)	0.0046 (2)	0.0097 (2)	0.0054 (2)
Cl4C	0.0394 (3)	0.0286 (3)	0.0347 (3)	−0.0078 (2)	0.0140 (2)	−0.0072 (2)
Cl5C	0.0435 (3)	0.0346 (3)	0.0368 (3)	0.0066 (2)	−0.0039 (3)	0.0066 (2)
Cl6C	0.0418 (3)	0.0253 (2)	0.0345 (3)	−0.0023 (2)	0.0052 (2)	−0.0022 (2)
C1C	0.0235 (9)	0.0347 (11)	0.0244 (10)	−0.0023 (8)	0.0058 (8)	0.0002 (8)
O1C	0.0276 (8)	0.0680 (13)	0.0246 (8)	−0.0053 (8)	0.0092 (7)	0.0033 (8)
N1C	0.0233 (8)	0.0335 (9)	0.0194 (8)	−0.0007 (7)	0.0057 (7)	0.0007 (7)
C2C	0.0226 (9)	0.0260 (9)	0.0215 (10)	−0.0006 (7)	0.0041 (7)	−0.0003 (7)
S1C	0.0224 (2)	0.0505 (3)	0.0238 (3)	0.0004 (2)	0.0072 (2)	−0.0006 (2)
N2C	0.0222 (8)	0.0347 (10)	0.0216 (9)	−0.0024 (7)	0.0056 (7)	0.0009 (7)
C11C	0.0208 (9)	0.0529 (15)	0.0233 (11)	0.0001 (9)	0.0065 (8)	0.0064 (10)
C12C	0.0276 (11)	0.077 (2)	0.0273 (12)	−0.0119 (12)	0.0107 (10)	−0.0077 (12)
C13C	0.0280 (13)	0.131 (4)	0.0284 (14)	−0.0114 (17)	0.0075 (11)	−0.0089 (18)
C14C	0.0267 (14)	0.166 (5)	0.0360 (17)	0.015 (2)	0.0073 (12)	0.029 (2)
C15C	0.0396 (16)	0.110 (3)	0.059 (2)	0.0335 (19)	0.0279 (15)	0.048 (2)
C16C	0.0315 (12)	0.0619 (18)	0.0450 (16)	0.0087 (12)	0.0177 (11)	0.0197 (13)
C21C	0.0239 (9)	0.0276 (10)	0.0201 (9)	−0.0021 (7)	0.0057 (7)	−0.0006 (7)
C22C	0.0264 (9)	0.0254 (10)	0.0232 (10)	−0.0001 (7)	0.0086 (8)	0.0002 (8)
C23C	0.0295 (10)	0.0299 (10)	0.0237 (10)	−0.0024 (8)	0.0099 (8)	−0.0003 (8)
C24C	0.0289 (10)	0.0338 (11)	0.0232 (10)	−0.0017 (8)	0.0042 (8)	−0.0018 (8)
C25C	0.0283 (10)	0.0311 (11)	0.0252 (11)	0.0017 (8)	0.0044 (8)	0.0025 (8)
C26C	0.0288 (10)	0.0263 (10)	0.0229 (10)	−0.0004 (8)	0.0060 (8)	−0.0002 (8)
C1L	0.0428 (14)	0.0476 (16)	0.0485 (17)	−0.0054 (12)	0.0050 (12)	−0.0016 (13)
Cl11	0.0525 (4)	0.0443 (4)	0.0376 (3)	0.0007 (3)	0.0036 (3)	0.0001 (3)
Cl12	0.0405 (3)	0.0493 (4)	0.0421 (4)	−0.0008 (3)	0.0108 (3)	0.0033 (3)
Cl13	0.0952 (7)	0.0521 (5)	0.0853 (7)	0.0218 (5)	0.0263 (6)	−0.0128 (5)
C2L	0.203 (7)	0.088 (4)	0.078 (3)	0.085 (4)	0.064 (4)	0.043 (3)
Cl21	0.0380 (3)	0.0529 (4)	0.0520 (4)	0.0034 (3)	0.0072 (3)	0.0148 (3)
Cl22	0.1235 (10)	0.0455 (5)	0.0873 (8)	0.0224 (6)	0.0191 (7)	−0.0095 (5)
Cl23	0.1051 (7)	0.0389 (4)	0.0421 (4)	−0.0021 (4)	−0.0074 (4)	0.0015 (3)

### Geometric parameters (Å, °)

**Table:**

Cl1—C12	1.732 (3)	Cl5B—C25B	1.731 (2)
Cl2—C16	1.742 (3)	Cl6B—C26B	1.723 (2)
Cl3—C22	1.719 (2)	C1B—O1B	1.216 (3)
Cl4—C23	1.734 (3)	C1B—N1B	1.376 (3)
Cl5—C25	1.725 (2)	C1B—C11B	1.511 (3)
Cl6—C26	1.722 (2)	N1B—C2B	1.387 (3)
C1—O1	1.215 (3)	N1B—H1B	0.83 (3)
C1—N1	1.378 (3)	C2B—N2B	1.343 (3)
C1—C11	1.506 (3)	C2B—S1B	1.669 (2)
N1—C2	1.388 (3)	N2B—C21B	1.417 (3)
N1—H1	0.80 (3)	N2B—H2B	0.78 (3)
C2—N2	1.338 (3)	C11B—C16B	1.390 (4)
C2—S1	1.670 (2)	C11B—C12B	1.395 (3)
N2—C21	1.414 (3)	C12B—C13B	1.394 (3)
N2—H2	0.78 (3)	C13B—C14B	1.386 (4)
C11—C12	1.392 (4)	C13B—H13B	0.9500
C11—C16	1.397 (4)	C14B—C15B	1.387 (4)
C12—C13	1.396 (3)	C14B—H14B	0.9500
C13—C14	1.375 (5)	C15B—C16B	1.389 (3)
C13—H13	0.9500	C15B—H15B	0.9500
C14—C15	1.380 (5)	C21B—C26B	1.395 (3)
C14—H14	0.9500	C21B—C22B	1.404 (3)
C15—C16	1.388 (3)	C22B—C23B	1.393 (3)
C15—H15	0.9500	C23B—C24B	1.383 (4)
C21—C22	1.397 (3)	C24B—C25B	1.388 (4)
C21—C26	1.400 (3)	C24B—H24B	0.9500
C22—C23	1.398 (3)	C25B—C26B	1.397 (3)
C23—C24	1.380 (3)	Cl1C—C12C	1.728 (4)
C24—C25	1.384 (3)	Cl2C—C16C	1.737 (4)
C24—H24	0.9500	Cl3C—C22C	1.722 (2)
C25—C26	1.399 (3)	Cl4C—C23C	1.729 (2)
Cl1A—C12A	1.745 (3)	Cl5C—C25C	1.726 (2)
Cl2A—C16A	1.732 (3)	Cl6C—C26C	1.718 (2)
Cl3A—C22A	1.728 (2)	C1C—O1C	1.216 (3)
Cl4A—C23A	1.727 (2)	C1C—N1C	1.377 (3)
Cl5A—C25A	1.730 (2)	C1C—C11C	1.501 (3)
Cl6A—C26A	1.721 (2)	N1C—C2C	1.391 (3)
C1A—O1A	1.218 (3)	N1C—H1C	0.86 (3)
C1A—N1A	1.377 (3)	C2C—N2C	1.338 (3)
C1A—C11A	1.505 (3)	C2C—S1C	1.671 (2)
N1A—C2A	1.391 (3)	N2C—C21C	1.419 (3)
N1A—H1A	0.84 (3)	N2C—H2C	0.77 (3)
C2A—N2A	1.332 (3)	C11C—C12C	1.393 (4)
C2A—S1A	1.671 (2)	C11C—C16C	1.395 (4)
N2A—C21A	1.418 (3)	C12C—C13C	1.405 (4)
N2A—H2A	0.75 (3)	C13C—C14C	1.379 (7)
C11A—C16A	1.388 (4)	C13C—H13C	0.9500
C11A—C12A	1.394 (4)	C14C—C15C	1.373 (7)
C12A—C13A	1.386 (4)	C14C—H14C	0.9500
C13A—C14A	1.389 (6)	C15C—C16C	1.385 (4)
C13A—H13A	0.9500	C15C—H15C	0.9500
C14A—C15A	1.364 (6)	C21C—C26C	1.394 (3)
C14A—H14A	0.9500	C21C—C22C	1.404 (3)
C15A—C16A	1.400 (4)	C22C—C23C	1.394 (3)
C15A—H15A	0.9500	C23C—C24C	1.387 (3)
C21A—C26A	1.395 (3)	C24C—C25C	1.386 (3)
C21A—C22A	1.395 (3)	C24C—H24C	0.9500
C22A—C23A	1.395 (3)	C25C—C26C	1.400 (3)
C23A—C24A	1.383 (3)	C1L—Cl13	1.734 (3)
C24A—C25A	1.389 (3)	C1L—Cl11	1.760 (3)
C24A—H24A	0.9500	C1L—Cl12	1.771 (3)
C25A—C26A	1.395 (3)	C1L—H1L	1.0000
Cl1B—C12B	1.739 (3)	C2L—Cl22	1.664 (6)
Cl2B—C16B	1.744 (3)	C2L—Cl23	1.679 (5)
Cl3B—C22B	1.721 (2)	C2L—Cl21	1.734 (4)
Cl4B—C23B	1.727 (2)	C2L—H2L	1.0000
			
O1—C1—N1	124.5 (2)	N2B—C2B—N1B	115.65 (19)
O1—C1—C11	121.3 (2)	N2B—C2B—S1B	124.06 (16)
N1—C1—C11	114.20 (19)	N1B—C2B—S1B	120.28 (16)
C1—N1—C2	127.01 (19)	C2B—N2B—C21B	122.98 (19)
C1—N1—H1	121 (2)	C2B—N2B—H2B	118 (3)
C2—N1—H1	112 (2)	C21B—N2B—H2B	119 (3)
N2—C2—N1	115.25 (19)	C16B—C11B—C12B	118.1 (2)
N2—C2—S1	124.24 (17)	C16B—C11B—C1B	120.2 (2)
N1—C2—S1	120.51 (17)	C12B—C11B—C1B	121.6 (2)
C2—N2—C21	123.69 (19)	C13B—C12B—C11B	121.2 (3)
C2—N2—H2	116 (3)	C13B—C12B—Cl1B	119.1 (2)
C21—N2—H2	120 (3)	C11B—C12B—Cl1B	119.66 (18)
C12—C11—C16	117.8 (2)	C14B—C13B—C12B	118.7 (3)
C12—C11—C1	121.0 (2)	C14B—C13B—H13B	120.6
C16—C11—C1	121.1 (2)	C12B—C13B—H13B	120.6
C11—C12—C13	121.2 (3)	C13B—C14B—C15B	121.7 (2)
C11—C12—Cl1	119.48 (19)	C13B—C14B—H14B	119.1
C13—C12—Cl1	119.3 (2)	C15B—C14B—H14B	119.1
C14—C13—C12	119.0 (3)	C14B—C15B—C16B	118.1 (3)
C14—C13—H13	120.5	C14B—C15B—H15B	120.9
C12—C13—H13	120.5	C16B—C15B—H15B	120.9
C13—C14—C15	121.7 (3)	C15B—C16B—C11B	122.1 (2)
C13—C14—H14	119.2	C15B—C16B—Cl2B	119.0 (2)
C15—C14—H14	119.2	C11B—C16B—Cl2B	118.85 (18)
C14—C15—C16	118.6 (3)	C26B—C21B—C22B	119.45 (19)
C14—C15—H15	120.7	C26B—C21B—N2B	120.8 (2)
C16—C15—H15	120.7	C22B—C21B—N2B	119.6 (2)
C15—C16—C11	121.7 (3)	C23B—C22B—C21B	119.5 (2)
C15—C16—Cl2	119.6 (2)	C23B—C22B—Cl3B	120.96 (18)
C11—C16—Cl2	118.68 (19)	C21B—C22B—Cl3B	119.45 (17)
C22—C21—C26	119.7 (2)	C24B—C23B—C22B	121.0 (2)
C22—C21—N2	120.4 (2)	C24B—C23B—Cl4B	118.59 (18)
C26—C21—N2	119.7 (2)	C22B—C23B—Cl4B	120.43 (19)
C21—C22—C23	119.6 (2)	C23B—C24B—C25B	119.6 (2)
C21—C22—Cl3	119.26 (17)	C23B—C24B—H24B	120.2
C23—C22—Cl3	121.09 (19)	C25B—C24B—H24B	120.2
C24—C23—C22	120.5 (2)	C24B—C25B—C26B	120.3 (2)
C24—C23—Cl4	118.72 (18)	C24B—C25B—Cl5B	118.83 (18)
C22—C23—Cl4	120.70 (19)	C26B—C25B—Cl5B	120.84 (19)
C23—C24—C25	120.1 (2)	C21B—C26B—C25B	120.1 (2)
C23—C24—H24	120.0	C21B—C26B—Cl6B	119.53 (17)
C25—C24—H24	120.0	C25B—C26B—Cl6B	120.37 (18)
C24—C25—C26	120.4 (2)	O1C—C1C—N1C	124.3 (2)
C24—C25—Cl5	118.72 (18)	O1C—C1C—C11C	121.2 (2)
C26—C25—Cl5	120.89 (19)	N1C—C1C—C11C	114.53 (19)
C25—C26—C21	119.6 (2)	C1C—N1C—C2C	126.88 (19)
C25—C26—Cl6	121.24 (18)	C1C—N1C—H1C	115.9 (19)
C21—C26—Cl6	119.14 (17)	C2C—N1C—H1C	116.9 (19)
O1A—C1A—N1A	124.5 (2)	N2C—C2C—N1C	115.53 (19)
O1A—C1A—C11A	121.1 (2)	N2C—C2C—S1C	124.22 (16)
N1A—C1A—C11A	114.40 (19)	N1C—C2C—S1C	120.25 (16)
C1A—N1A—C2A	126.97 (19)	C2C—N2C—C21C	123.16 (19)
C1A—N1A—H1A	114.3 (19)	C2C—N2C—H2C	113 (2)
C2A—N1A—H1A	118.7 (19)	C21C—N2C—H2C	123 (2)
N2A—C2A—N1A	115.45 (19)	C12C—C11C—C16C	118.3 (2)
N2A—C2A—S1A	124.23 (16)	C12C—C11C—C1C	121.9 (3)
N1A—C2A—S1A	120.32 (16)	C16C—C11C—C1C	119.7 (2)
C2A—N2A—C21A	123.26 (19)	C11C—C12C—C13C	120.6 (3)
C2A—N2A—H2A	117 (3)	C11C—C12C—Cl1C	119.5 (2)
C21A—N2A—H2A	120 (3)	C13C—C12C—Cl1C	119.8 (3)
C16A—C11A—C12A	117.9 (2)	C14C—C13C—C12C	118.9 (4)
C16A—C11A—C1A	121.2 (2)	C14C—C13C—H13C	120.6
C12A—C11A—C1A	120.8 (2)	C12C—C13C—H13C	120.6
C13A—C12A—C11A	122.0 (3)	C15C—C14C—C13C	121.7 (3)
C13A—C12A—Cl1A	119.2 (3)	C15C—C14C—H14C	119.1
C11A—C12A—Cl1A	118.8 (2)	C13C—C14C—H14C	119.1
C12A—C13A—C14A	118.2 (3)	C14C—C15C—C16C	119.0 (4)
C12A—C13A—H13A	120.9	C14C—C15C—H15C	120.5
C14A—C13A—H13A	120.9	C16C—C15C—H15C	120.5
C15A—C14A—C13A	121.7 (3)	C15C—C16C—C11C	121.6 (3)
C15A—C14A—H14A	119.1	C15C—C16C—Cl2C	119.7 (3)
C13A—C14A—H14A	119.1	C11C—C16C—Cl2C	118.8 (2)
C14A—C15A—C16A	119.2 (3)	C26C—C21C—C22C	119.56 (19)
C14A—C15A—H15A	120.4	C26C—C21C—N2C	120.9 (2)
C16A—C15A—H15A	120.4	C22C—C21C—N2C	119.38 (19)
C11A—C16A—C15A	121.1 (3)	C23C—C22C—C21C	119.7 (2)
C11A—C16A—Cl2A	119.27 (19)	C23C—C22C—Cl3C	121.21 (17)
C15A—C16A—Cl2A	119.6 (3)	C21C—C22C—Cl3C	119.13 (16)
C26A—C21A—C22A	119.9 (2)	C24C—C23C—C22C	120.7 (2)
C26A—C21A—N2A	120.2 (2)	C24C—C23C—Cl4C	118.29 (17)
C22A—C21A—N2A	119.7 (2)	C22C—C23C—Cl4C	120.97 (18)
C23A—C22A—C21A	119.6 (2)	C25C—C24C—C23C	119.7 (2)
C23A—C22A—Cl3A	121.07 (18)	C25C—C24C—H24C	120.1
C21A—C22A—Cl3A	119.28 (16)	C23C—C24C—H24C	120.1
C24A—C23A—C22A	120.5 (2)	C24C—C25C—C26C	120.3 (2)
C24A—C23A—Cl4A	118.36 (17)	C24C—C25C—Cl5C	118.67 (17)
C22A—C23A—Cl4A	121.08 (18)	C26C—C25C—Cl5C	120.99 (18)
C23A—C24A—C25A	119.9 (2)	C21C—C26C—C25C	120.0 (2)
C23A—C24A—H24A	120.1	C21C—C26C—Cl6C	119.50 (16)
C25A—C24A—H24A	120.1	C25C—C26C—Cl6C	120.50 (17)
C24A—C25A—C26A	120.2 (2)	Cl13—C1L—Cl11	111.14 (17)
C24A—C25A—Cl5A	118.63 (18)	Cl13—C1L—Cl12	111.4 (2)
C26A—C25A—Cl5A	121.11 (19)	Cl11—C1L—Cl12	110.41 (18)
C25A—C26A—C21A	119.8 (2)	Cl13—C1L—H1L	107.9
C25A—C26A—Cl6A	121.06 (19)	Cl11—C1L—H1L	107.9
C21A—C26A—Cl6A	119.15 (17)	Cl12—C1L—H1L	107.9
O1B—C1B—N1B	124.7 (2)	Cl22—C2L—Cl23	118.2 (3)
O1B—C1B—C11B	121.3 (2)	Cl22—C2L—Cl21	116.2 (4)
N1B—C1B—C11B	114.02 (19)	Cl23—C2L—Cl21	115.5 (3)
C1B—N1B—C2B	127.06 (19)	Cl22—C2L—H2L	100.7
C1B—N1B—H1B	120 (2)	Cl23—C2L—H2L	100.7
C2B—N1B—H1B	113 (2)	Cl21—C2L—H2L	100.7
			
O1—C1—N1—C2	−2.3 (4)	O1B—C1B—N1B—C2B	8.1 (4)
C11—C1—N1—C2	177.2 (2)	C11B—C1B—N1B—C2B	−170.0 (2)
C1—N1—C2—N2	−0.9 (4)	C1B—N1B—C2B—N2B	−1.8 (3)
C1—N1—C2—S1	179.49 (19)	C1B—N1B—C2B—S1B	178.02 (19)
N1—C2—N2—C21	−178.7 (2)	N1B—C2B—N2B—C21B	166.2 (2)
S1—C2—N2—C21	0.9 (3)	S1B—C2B—N2B—C21B	−13.6 (3)
O1—C1—C11—C12	−81.7 (3)	O1B—C1B—C11B—C16B	−96.7 (3)
N1—C1—C11—C12	98.7 (3)	N1B—C1B—C11B—C16B	81.5 (3)
O1—C1—C11—C16	93.4 (3)	O1B—C1B—C11B—C12B	78.7 (3)
N1—C1—C11—C16	−86.1 (3)	N1B—C1B—C11B—C12B	−103.1 (3)
C16—C11—C12—C13	1.0 (4)	C16B—C11B—C12B—C13B	−1.3 (4)
C1—C11—C12—C13	176.3 (2)	C1B—C11B—C12B—C13B	−176.8 (2)
C16—C11—C12—Cl1	−178.02 (19)	C16B—C11B—C12B—Cl1B	177.23 (18)
C1—C11—C12—Cl1	−2.7 (3)	C1B—C11B—C12B—Cl1B	1.7 (3)
C11—C12—C13—C14	−0.7 (4)	C11B—C12B—C13B—C14B	0.3 (4)
Cl1—C12—C13—C14	178.3 (2)	Cl1B—C12B—C13B—C14B	−178.3 (2)
C12—C13—C14—C15	−0.1 (4)	C12B—C13B—C14B—C15B	0.7 (4)
C13—C14—C15—C16	0.5 (4)	C13B—C14B—C15B—C16B	−0.6 (4)
C14—C15—C16—C11	−0.1 (4)	C14B—C15B—C16B—C11B	−0.4 (4)
C14—C15—C16—Cl2	−178.8 (2)	C14B—C15B—C16B—Cl2B	177.4 (2)
C12—C11—C16—C15	−0.6 (4)	C12B—C11B—C16B—C15B	1.4 (4)
C1—C11—C16—C15	−175.9 (2)	C1B—C11B—C16B—C15B	176.9 (2)
C12—C11—C16—Cl2	178.10 (18)	C12B—C11B—C16B—Cl2B	−176.42 (18)
C1—C11—C16—Cl2	2.8 (3)	C1B—C11B—C16B—Cl2B	−0.9 (3)
C2—N2—C21—C22	88.3 (3)	C2B—N2B—C21B—C26B	112.3 (3)
C2—N2—C21—C26	−96.7 (3)	C2B—N2B—C21B—C22B	−72.4 (3)
C26—C21—C22—C23	−1.2 (3)	C26B—C21B—C22B—C23B	−0.2 (3)
N2—C21—C22—C23	173.8 (2)	N2B—C21B—C22B—C23B	−175.5 (2)
C26—C21—C22—Cl3	178.82 (18)	C26B—C21B—C22B—Cl3B	177.49 (17)
N2—C21—C22—Cl3	−6.2 (3)	N2B—C21B—C22B—Cl3B	2.1 (3)
C21—C22—C23—C24	1.5 (4)	C21B—C22B—C23B—C24B	−0.7 (3)
Cl3—C22—C23—C24	−178.55 (19)	Cl3B—C22B—C23B—C24B	−178.28 (19)
C21—C22—C23—Cl4	−176.41 (18)	C21B—C22B—C23B—Cl4B	177.96 (17)
Cl3—C22—C23—Cl4	3.5 (3)	Cl3B—C22B—C23B—Cl4B	0.4 (3)
C22—C23—C24—C25	−0.3 (4)	C22B—C23B—C24B—C25B	0.9 (4)
Cl4—C23—C24—C25	177.67 (19)	Cl4B—C23B—C24B—C25B	−177.72 (19)
C23—C24—C25—C26	−1.2 (4)	C23B—C24B—C25B—C26B	−0.4 (4)
C23—C24—C25—Cl5	−179.95 (19)	C23B—C24B—C25B—Cl5B	178.89 (19)
C24—C25—C26—C21	1.4 (3)	C22B—C21B—C26B—C25B	0.7 (3)
Cl5—C25—C26—C21	−179.82 (17)	N2B—C21B—C26B—C25B	176.0 (2)
C24—C25—C26—Cl6	−178.41 (18)	C22B—C21B—C26B—Cl6B	−178.78 (17)
Cl5—C25—C26—Cl6	0.3 (3)	N2B—C21B—C26B—Cl6B	−3.4 (3)
C22—C21—C26—C25	−0.2 (3)	C24B—C25B—C26B—C21B	−0.5 (4)
N2—C21—C26—C25	−175.3 (2)	Cl5B—C25B—C26B—C21B	−179.70 (18)
C22—C21—C26—Cl6	179.63 (18)	C24B—C25B—C26B—Cl6B	179.03 (19)
N2—C21—C26—Cl6	4.6 (3)	Cl5B—C25B—C26B—Cl6B	−0.2 (3)
O1A—C1A—N1A—C2A	−1.6 (4)	O1C—C1C—N1C—C2C	10.0 (4)
C11A—C1A—N1A—C2A	177.8 (2)	C11C—C1C—N1C—C2C	−169.2 (2)
C1A—N1A—C2A—N2A	1.2 (3)	C1C—N1C—C2C—N2C	−2.2 (3)
C1A—N1A—C2A—S1A	−178.15 (19)	C1C—N1C—C2C—S1C	177.26 (19)
N1A—C2A—N2A—C21A	−179.5 (2)	N1C—C2C—N2C—C21C	165.8 (2)
S1A—C2A—N2A—C21A	−0.2 (3)	S1C—C2C—N2C—C21C	−13.7 (3)
O1A—C1A—C11A—C16A	−87.3 (3)	O1C—C1C—C11C—C12C	85.8 (3)
N1A—C1A—C11A—C16A	93.3 (3)	N1C—C1C—C11C—C12C	−95.0 (3)
O1A—C1A—C11A—C12A	87.6 (3)	O1C—C1C—C11C—C16C	−90.5 (3)
N1A—C1A—C11A—C12A	−91.8 (3)	N1C—C1C—C11C—C16C	88.6 (3)
C16A—C11A—C12A—C13A	−0.6 (4)	C16C—C11C—C12C—C13C	−0.3 (4)
C1A—C11A—C12A—C13A	−175.6 (2)	C1C—C11C—C12C—C13C	−176.7 (2)
C16A—C11A—C12A—Cl1A	177.91 (19)	C16C—C11C—C12C—Cl1C	178.1 (2)
C1A—C11A—C12A—Cl1A	2.8 (3)	C1C—C11C—C12C—Cl1C	1.7 (3)
C11A—C12A—C13A—C14A	0.6 (4)	C11C—C12C—C13C—C14C	0.3 (4)
Cl1A—C12A—C13A—C14A	−177.9 (2)	Cl1C—C12C—C13C—C14C	−178.1 (2)
C12A—C13A—C14A—C15A	−0.5 (5)	C12C—C13C—C14C—C15C	0.0 (5)
C13A—C14A—C15A—C16A	0.4 (5)	C13C—C14C—C15C—C16C	−0.3 (5)
C12A—C11A—C16A—C15A	0.4 (4)	C14C—C15C—C16C—C11C	0.2 (5)
C1A—C11A—C16A—C15A	175.5 (2)	C14C—C15C—C16C—Cl2C	−179.8 (2)
C12A—C11A—C16A—Cl2A	−178.72 (19)	C12C—C11C—C16C—C15C	0.1 (4)
C1A—C11A—C16A—Cl2A	−3.7 (3)	C1C—C11C—C16C—C15C	176.5 (2)
C14A—C15A—C16A—C11A	−0.4 (4)	C12C—C11C—C16C—Cl2C	−179.93 (19)
C14A—C15A—C16A—Cl2A	178.8 (2)	C1C—C11C—C16C—Cl2C	−3.5 (3)
C2A—N2A—C21A—C26A	91.8 (3)	C2C—N2C—C21C—C26C	104.0 (3)
C2A—N2A—C21A—C22A	−92.9 (3)	C2C—N2C—C21C—C22C	−80.7 (3)
C26A—C21A—C22A—C23A	−0.7 (3)	C26C—C21C—C22C—C23C	−0.6 (3)
N2A—C21A—C22A—C23A	−175.9 (2)	N2C—C21C—C22C—C23C	−176.0 (2)
C26A—C21A—C22A—Cl3A	179.80 (17)	C26C—C21C—C22C—Cl3C	179.09 (17)
N2A—C21A—C22A—Cl3A	4.5 (3)	N2C—C21C—C22C—Cl3C	3.7 (3)
C21A—C22A—C23A—C24A	1.2 (3)	C21C—C22C—C23C—C24C	0.9 (3)
Cl3A—C22A—C23A—C24A	−179.30 (18)	Cl3C—C22C—C23C—C24C	−178.75 (18)
C21A—C22A—C23A—Cl4A	−179.68 (17)	C21C—C22C—C23C—Cl4C	179.53 (17)
Cl3A—C22A—C23A—Cl4A	−0.2 (3)	Cl3C—C22C—C23C—Cl4C	−0.2 (3)
C22A—C23A—C24A—C25A	−0.7 (4)	C22C—C23C—C24C—C25C	−0.5 (4)
Cl4A—C23A—C24A—C25A	−179.82 (19)	Cl4C—C23C—C24C—C25C	−179.13 (18)
C23A—C24A—C25A—C26A	−0.4 (4)	C23C—C24C—C25C—C26C	−0.3 (4)
C23A—C24A—C25A—Cl5A	178.24 (19)	C23C—C24C—C25C—Cl5C	178.69 (18)
C24A—C25A—C26A—C21A	0.9 (4)	C22C—C21C—C26C—C25C	−0.2 (3)
Cl5A—C25A—C26A—C21A	−177.70 (18)	N2C—C21C—C26C—C25C	175.1 (2)
C24A—C25A—C26A—Cl6A	−178.40 (19)	C22C—C21C—C26C—Cl6C	−179.91 (17)
Cl5A—C25A—C26A—Cl6A	3.0 (3)	N2C—C21C—C26C—Cl6C	−4.6 (3)
C22A—C21A—C26A—C25A	−0.4 (3)	C24C—C25C—C26C—C21C	0.6 (4)
N2A—C21A—C26A—C25A	174.9 (2)	Cl5C—C25C—C26C—C21C	−178.33 (18)
C22A—C21A—C26A—Cl6A	178.94 (17)	C24C—C25C—C26C—Cl6C	−179.64 (19)
N2A—C21A—C26A—Cl6A	−5.8 (3)	Cl5C—C25C—C26C—Cl6C	1.4 (3)

### Hydrogen-bond geometry (Å, °)

**Table:**

*D*—H···*A*	*D*—H	H···*A*	*D*···*A*	*D*—H···*A*
N1—H1···S1B	0.80 (3)	2.60 (3)	3.362 (2)	160 (3)
N2—H2···O1	0.78 (3)	1.96 (3)	2.621 (2)	142 (3)
N2—H2···Cl4C^i^	0.78 (3)	2.94 (3)	3.539 (2)	135 (3)
N1A—H1A···S1C	0.84 (3)	2.55 (3)	3.381 (2)	170 (3)
N2A—H2A···O1A	0.75 (3)	2.00 (3)	2.625 (3)	141 (3)
N2A—H2A···Cl4A^ii^	0.75 (3)	2.90 (3)	3.478 (2)	136 (3)
N1B—H1B···S1	0.83 (3)	2.63 (3)	3.425 (2)	162 (3)
N2B—H2B···O1B	0.78 (3)	2.01 (3)	2.639 (2)	138 (3)
N1C—H1C···S1A	0.86 (3)	2.57 (3)	3.416 (2)	168 (3)
N2C—H2C···O1C	0.77 (3)	1.97 (3)	2.629 (3)	144 (3)

Symmetry codes: (i) *x*+1/2, −*y*+1/2, *z*+1/2; (ii) −*x*+1, −*y*+1, −*z*+1.
